# Clinical Predictors of Early Trial Discontinuation for Patients Participating in Phase I Clinical Trials in Oncology

**DOI:** 10.3390/cancers13102304

**Published:** 2021-05-11

**Authors:** Joeri A. J. Douma, Laurien M. Buffart, Ramy Sedhom, Mariette Labots, Willemien C. Menke-van der Houven van Oordt, Mikkjal Skardhamar, Anthony De Felice, Esther Lee, Divya Dharmaraj, Nilofer S. Azad, Michael A. Carducci, Henk M. W. Verheul

**Affiliations:** 1 Cancer Center Amsterdam, Amsterdam University Medical Center, Department of Medical Oncology, Vrije Universiteit Amsterdam, 1081 HV Amsterdam, The Netherlands; j.douma@nki.nl (J.A.J.D.); m.labots@amsterdamumc.nl (M.L.); c.menke@amsterdamumc.nl (W.C.M.-v.d.H.v.O.); m.skardhamar@amsterdamumc.nl (M.S.); 2 Department of Physiology, Radboudumc, 6525 GA Nijmegen, The Netherlands; Laurien.buffart@radboudumc.nl; 3 Sidney Kimmel Comprehensive Cancer Center at Johns Hopkins, Baltimore, MD 21231, USA; rsedhom1@jhmi.edu (R.S.); adefeli3@jhu.edu (A.D.F.); elee138@jhu.edu (E.L.); ddharma1@jhu.edu (D.D.); Nazad2@jhmi.edu (N.S.A.); Carducci@jhmi.edu (M.A.C.); 4 Department of Medical Oncology, Radboudumc, 6525 GA Nijmegen, The Netherlands

**Keywords:** phase I trial, drug development, early trial discontinuation, clinical predictors, hyponatremia

## Abstract

**Simple Summary:**

About 20% of patients with cancer who participate in a phase I clinical trial discontinue the trial early. Early trial discontinuation is undesirable both for the wellbeing of the patient as well as for the trial efficiency and the development of new anticancer drugs. We investigated which clinical predictors at baseline were significantly associated with early trial discontinuation of patients with cancer who participated in phase I clinical trials. The clinical predictors which were identified in this study were hyponatremia, elevated alkaline phosphatase level, performance score of 1 or higher and opioid use. Hyponatremia especially, which was the strongest predictor, should be considered to be used as an additional eligibility criterion in order to reduce the early trial discontinuation of patients with cancer who participate in phase I clinical trials.

**Abstract:**

Despite stringent eligibility criteria for trial participation, early discontinuation often occurs in phase I trials. To better identify patients unlikely to benefit from phase I trials, we investigated predictors for early trial discontinuation. Data from 415 patients with solid tumors who participated in 66 trials were pooled for the current analysis. Early trial discontinuation was defined as (i) trial discontinuation within 28 days after start of treatment or (ii) discontinuation before administration of the first dosage in eligible patients. Multilevel logistic regression analyses were conducted to identify predictors for early trial discontinuation. Eighty-two participants (20%) demonstrated early trial discontinuation. Baseline sodium level below the lower limit of normal (OR = 2.95, 95%CI = 1.27–6.84), elevated alkaline phosphatase level > 2.5 times the upper limit of normal (OR = 2.72, 95%CI = 1.49–4.99), performance score ≥ 1 (OR = 2.07, 95%CI = 1.03–4.19) and opioid use (OR = 1.82, 95%CI = 1.07–3.08) were independent predictors for early trial discontinuation. Almost 50% of the patients with hyponatremia and all four patients in whom all four predictors were present together discontinued the trial early. Hyponatremia, elevated alkaline phosphatase level, performance score ≥ 1 and opioid use were identified as significant predictors for early trial discontinuation. Hyponatremia was the strongest predictor and deserves consideration for inclusion in eligibility criteria for future trials.

## 1. Introduction

Phase I trials in oncology are essential for drug development and evaluation of novel treatment strategies [1]. Traditionally, phase I trials are designed to test the safety and side effects of a new drug or treatment strategy, with the aim of establishing the recommended dose for subsequent trials [2]. Since patients who are referred for phase I trial participation are usually heavily pretreated, the eligibility criteria for these trials are stringent to minimize the potential harm of study treatment. To be eligible for participation for most trials, patients must have an adequate performance status (PS) and organ function and a minimal life expectancy of 3 months without treatment [1,2,3]. As a result of these strict criteria, only 30% of referred patients are eligible, with an insufficient PS being the main reason for ineligibility [4,5]. 

Despite the stringent selection, previous studies revealed that 15% of participants in phase I trials discontinue within three weeks [6]. Patients who discontinue the trial early are not likely to benefit from study treatment. Moreover, participation in a phase I trial may yield additional burdens for patients due to possible adverse effects, multiple (long) visits at the clinic, invasive procedures and sometimes even extra financial costs [7,8,9]. In addition, study outcomes are negatively influenced by high rates of patients discontinuing the trial early. Approximately 70% of early trial discontinuation is due to a deteriorating condition caused by progressive disease and/or concomitant medical events that are not related to the study treatment [6]. Furthermore, patients who discontinue trial participation during the evaluation period because of non-drug-related events need to be replaced for evaluation of safety and dose limiting toxicity (DLT). The higher accrual necessary for adequate evaluation is detrimental to the efficient conduct of trials [5,6]. 

Prevention of early dropout may benefit the conduct and outcome of a trial [2]. In two previous studies, predictors for early trial discontinuation in participants of phase I trials conducted before 2010 have been reported [6,10]. The results of these studies were inconsistent, with the exception of PS and serum alkaline phosphatase, which were consistently identified as predictors. In addition, these studies are relatively old and did not include newer treatment strategies such as vaccines, radiopharmaceuticals and immunotherapy. We here investigated clinical or patient-related predictors of early trial discontinuation of participants of various types of phase I trials, which could be used as additional eligibility criteria across trials in the future. Furthermore, we studied the rate of 90-day mortality, which is a frequently used eligibility criterion in phase I trials [3], both in patients with and without early trial discontinuation to emphasize that early trial discontinuation is a relevant measure. 

## 2. Materials and Methods

### 2.1. Study Design and Population

For the current retrospective analysis, we pooled data from patients with solid tumors who participated in phase I trials at the Amsterdam UMC, Vrije Universiteit Amsterdam, in Amsterdam, The Netherlands, and at the Sidney Kimmel Comprehensive Cancer Center at Johns Hopkins in Baltimore, US. The phase I trials were conducted between January 2013 and January 2019. All data were collected in the context of the phase I trial and were retrieved from the electronic health records of the hospital. All study protocols were approved by Institutional Review Boards prior to patient recruitment and conducted in accordance with the International Conference on Harmonization E6 Guidelines for Good Clinical Practice. All patients provided written informed consent before participation in the phase I trials. 

### 2.2. Outcome

Early trial discontinuation was defined as (i) trial discontinuation within 28 days of administration of the first dosage of the investigated drug or (ii) discontinuation before administration of the first dosage in patients who were found to be eligible after the screening process. Patients who met these criteria would not have had a chance to benefit from the treatment, although they may have experienced toxicity or complications from treatment or investigations during the screening process. Ninety-day mortality was defined as (i) death from any cause within 90 days of administration of the first dosage of the investigated drug or (ii) death from any cause before administration of the first dosage in patients who were found to be eligible after screening. 

### 2.3. Potential Predictors

Potential predictors and cut-offs were defined before reviewing the data and selected based on their previously reported association with early trial discontinuation, 90-day mortality, overall survival or clinical relevance in participants of phase I oncology trials [6,10,11]. The following baseline characteristics were selected: opioid use, number of metastatic sites [6], body mass index (BMI), ECOG/WHO PS [6,10], history of thromboembolism [12], hemoglobin [13], platelet count [12], white blood cell count [6], lymphocytes [6], absolute neutrophil count, neutrophils-to-lymphocytes (NTL) ratio [14], serum sodium [15,16], creatinine clearance [10], serum albumin [10], serum alkaline phosphatase [10], serum aspartate aminotransferase [10], serum alanine aminotransferase [10], serum lactate dehydrogenase [6] and the Charlson Comorbidity Index [17]. BMI was calculated from measurements of body height and weight (body weight/height^2^, kg/m^2^). Creatinine clearance was estimated by using the Chronic Kidney Disease Epidemiology Collaboration formula [18]. The NTL ratio was calculated by dividing the neutrophils by the lymphocytes. All of the above predictors were dichotomized according to the cut-off values that were found in the literature to facilitate interpretability and use in clinical practice (Table 1).

### 2.4. Statistical Analysis

Univariable and multivariable logistic regression analyses were conducted to identify factors that were significantly associated with early trial discontinuation. Potentially relevant variables identified from the univariable analysis were checked for multicolinearity (r ≥ 0.60). A stepwise forward selection procedure was used to build the multivariable regression model, starting with the variable that was most strongly associated with the outcome in the univariable regression model. Subsequently, the next strongest variable was selected after controlling for the first variable. This procedure was repeated until no variables with an association with the outcome at a significance level of *p* < 0.05 could be added to the model. A random intercept was added to the model to take into account the clustering of patients within studies. We also checked whether the hospital where the trial was performed (Amsterdam UMC, Vrije Universiteit Amsterdam versus the Sidney Kimmel Comprehensive Cancer Center at Johns Hopkins), the type of therapy (immunotherapy versus other types of treatment) or the type of the trial (phase I versus phase I/II) were non-patient-related predictors of early trial discontinuation, but none of the associations were statistically significant. As one study (NCT02058901) provided most of the data, we performed sensitivity analyses on the data excluding that study. Descriptive statistics and a chi-squared test were used to compare the rate of 90-day mortality both in patients with and without early trial discontinuation. The odds ratio (OR) and 95% confidence interval (CI) of the models were reported. Crosstabs were generated to present the proportion of patients with early trial discontinuation and separately for all the significant predictors identified in the multivariable analyses. Subsequently, we calculated the positive predictive value for the combination of predictors. Analyses were conducted with SPSS version 22 and RStudio version 3.4.2.

## 3. Results

Data from 415 patients recruited from 66 phase I trials were analyzed. In 21% of the trials, immunotherapy alone was investigated, in 20% immunotherapy in combination with targeted therapy, in 18% targeted therapy alone, in 16% targeted therapy in combination with cytotoxic therapy and in the remaining 25% other types of drugs or combinations of drugs were investigated (Appendix A). The mean age of the patients was 61 (standard deviation (SD) = 11) years, half of patients were women and 69% had a PS of 1 (Table 1). In total, 82 patients (20%) met the criteria for early trial discontinuation. Five patients (5/82 or 6%) discontinued trial participation before administration of the first dosage. For the patients who received the first administration of the drug and discontinued the trial early, the average time to trial discontinuation was 17 days (SD = 7). Thirty-six patients (44%) discontinued the trial early due to progressive disease, 15 (18%) due to physical deterioration, 10 patients (12%) due to toxicity that was not dose limiting and 10 patients (12%) discontinued the trial upon their own request (Table 2). 

In the multivariable model, a reduced serum sodium level (OR = 2.95, 95%CI = 1.27–6.84), an elevated alkaline phosphatase level above 2.5 times the upper limit of normal (ULN) (OR = 2.72, 95%CI = 1.49–4.99), a PS of ≥1 (OR = 2.07, 95%CI = 1.03–4.19) and opioid use (OR = 1.82, 95%CI = 1.07–3.08) were significantly and independently associated with early trial discontinuation (Table 3). The positive predictive value for early trial discontinuation was 46% for hyponatremia, 39% for elevated alkaline phosphatase level, 38% for opioid use and 31% for PS ≥ 1. Hyponatremia was the predictor with the highest positive predictive value, based on the fact that 13 (46%) of the 28 patients with hyponatremia discontinued the trial early. The median (interquartile range) sodium level was 139 (137–141) mmol/L, with a lower limit of normal of 135 mmol/L in both hospitals. Furthermore, all four predictors were present in four patients. The positive predictive value in this situation was 100%. Sensitivity analyses on the data excluding the trial with the most participants yielded comparable results.

In total, 59% of the patients who discontinued early died within 90 days of starting the trial compared to 13% of the patients who did not discontinue the trial early (*p* < 0.001). 

## 4. Discussion

Early trial discontinuation in phase I trials is an important and common problem, for which a rate of 20% was found in this study. Hyponatremia, PS ≥ 1, opioid use and elevated alkaline phosphatase level were identified as significant independent predictors for early trial discontinuation amongst patients who participated in phase I trials. Furthermore, the rate of 90-day mortality was significantly higher in patients who discontinued the trial early than in patients who did not.

Importantly, one novel finding is that hyponatremia was identified as a significant predictor for early trial discontinuation. In earlier studies, hyponatremia has not been identified or investigated as a potential predictor for early trial discontinuation [6,10] but was has been to be predictive for 90-day mortality and overall survival in patients participating in phase I oncology trials [15,16] and for overall survival in patients with breast, colorectal and lung cancer [19]. In an ancillary analysis, we found that hyponatremia was also significantly associated with 90-day mortality (OR = 3.62, 95%CI = 1.63–8.02). None of the included phase I trials in this study, nor any of the cancer trials examined in a review [3], used serum sodium level as eligibility criterion. There are several possible explanations why hyponatremia could be predictive for early trial discontinuation. Hyponatremia can be caused by the syndrome of inappropriate anti-diuretic hormone (SIADH) (due to cancer, pain or co-medication), by hypo- or hypervolemia and, although rare, by reduced salt intake [19]. We could not identify any corresponding clinical etiology (e.g., comorbidity, use of painkillers, co-medication) for the hyponatremia in the patients from our trials. Unfortunately, extensive diagnostics (e.g., MRI scan of the brain) to determine the cause of the hyponatremia were not performed in most patients. However, hyponatremia may also reflect a more advanced, otherwise undetected, stage of cancer in general. Further exploration of the etiology and the optimal cut-off value of hyponatremia should be considered in future studies and, more importantly, the role of hyponatremia as an eligibility criterion should be further investigated and might be used in future phase I trials. In the current study, lowering the cut-off value to the threshold of grade 1 hyponatremia (sodium < 130 mmol/L), conforming to the Common Terminology Criteria for Adverse Events (CTCAE), did not result in a clinically relevant improvement in the positive predictive value.

The results of the current study also indicated that opioid use was predictive for early trial discontinuation. The use of opioids has not been investigated earlier as a predictor for early trial discontinuation or mortality in participants in phase I trials. However, opioid use is an expression of cancer-related pain, which was found to be a significant predictor of overall survival in a systematic review and in meta-analysis in patients with different types of cancer [20,21]. Both PS and an elevated alkaline phosphatase level were identified as significant predictors of early trial discontinuation. This is in line with two earlier studies, in which elevated alkaline phosphatase level and PS were identified as significant predictors for early trial discontinuation in phase I trial participants [6,10]. 

Almost 60% of the patients with early trial discontinuation died within 90 days. Both early trial discontinuation and 90-day mortality are most likely caused by early progression, study-related toxicities, other complications or true inefficacy of the treatment in combination with an incorrectly estimated life expectancy. Patients who will die within 90 days should not be included in a trial [3] and should be offered advanced care planning, aggressive symptom management and discussion of end of life [22,23]. A better selection of patients to be included in a trial might reduce both early trial discontinuation as well as 90-day mortality. 

A possible limitation of this study might have been the inclusion of heterogeneous trials that investigated newer therapies like vaccines, kinase inhibitors and radiopharmaceuticals, as well as cytotoxic agents. On the other hand, the heterogeneity may enhance the generalizability of the results to other phase I trials. Furthermore, the current study, with a sample size of 415 and 82 events, is one of the largest studies evaluating predictors of early trial discontinuation and made it possible to study up to 8 predictors in the multivariable analysis. 

In general, phase I trial participation offers intensified care for cancer-related symptoms, which can improve quality of life and may even improve survival [24,25,26], regardless of the phase I study drugs administered. This study identified two new predictors for early trial discontinuation in patients with cancer participating in phase I trials. Notably, a high positive predictive value of the predictor is important in this setting because participation could be harmful to patients, but unnecessary exclusion for a potential beneficial drug is also undesirable. The positive predictive value of hyponatremia is quite high and has clinical relevance, but sodium level is currently not used as an eligibility criterion. Previous studies have shown that only 15% of patients experience a clinical benefit from experimental treatment in phase 1 trials at 6 months [27,28]. This proportion is likely substantially lower in patients with early trial discontinuation. Due to the low chance of clinical benefit in combination with the potential harm of participation in a phase I clinical trial, a positive predictive value of around 50% for early trial discontinuation supports the use of hyponatremia as an exclusion criterion and to exclude these patients from participation in phase I trials. 

The results of this study indicate that hyponatremia might be useful for trial selection and we propose that this criterion deserves further investigation. Changes to the conduct of early phase trials should be carefully considered because they potentially have far-reaching effects [29]. Unnecessary restrictive eligibility criteria should be avoided to maintain generalizability to the patients who will ultimately be treated with the investigated drugs [30]. More stringent eligibility criteria are warranted if research indicates that patients have a high risk for an adverse outcome when participating in the study, for example early trial discontinuation. On the other hand, some eligibility criteria (like brain metastases, HIV infection and concurrent malignancies) that are not likely to protect the safety of trial participants should be loosened or abandoned [30]. As patients with hyponatremia have a high risk of early trial discontinuation, we hypothesize that adjustment of the eligibility criteria with the addition of hyponatremia might contribute to a better selection of patients. Therefore, prospective validation of our results is warranted to determine whether adjustments of eligibility criteria are needed and to investigate whether the threshold for hyponatremia could be lowered. In the meanwhile, clinicians should be alerted that patients with hyponatremia, opioid use, PS ≥ 1 and an elevated alkaline phosphatase level are at risk for an unfavorable outcome and should be closely monitored, with appropriate initiation of supportive or palliative care if needed.

## 5. Conclusions

In conclusion, hyponatremia, opioid use, PS ≥ 1 and an elevated alkaline phosphatase level were identified as predictors for early trial discontinuation amongst patients with cancer participating in phase I trials. Hyponatremia was the strongest predictor with a positive predictive value of 46% and discussion should be centered on this as a possible exclusion criterion in future phase I trials in oncology. Furthermore, the rate of 90-day mortality was significantly higher in patients who discontinued the trial early than in patients who did not. 

## Figures and Tables

**Table 1:** Baseline patient characteristics (*n* = 415).

Characteristic	Statistics
Age, mean (SD) years	61 (11)
Sex, *n* (%) women	204 (49)
Recruited in the Netherlands, *n* (%)	154 (37)
Primary tumor, *n* (%)	
Gastrointestinal	207 (50)
Genitourinary	42 (10)
Lung cancer	32 (8)
Skin and soft tissue cancer	31 (7)
Breast cancer	28 (7)
Gynecological	19 (5)
Head and neck	18 (4)
Glioblastoma	17 (4)
Neuroendocrine carcinoma	8 (2)
Others	13 (3)
ECOG/WHO performance status, *n* (%) ^a^	
0	118 (29)
1	285 (69)
>1	5 (1)
BMI, mean (SD) (kg/m^2^)	26 (5)
BMI < 18.5, *n* (%) ^b^	20 (5)
Opioid use, *n* (%) ^c^	177 (43)
Three or more metastatic sites, *n* (%)	118 (77)
Any comorbidity other than primary malignancy, *n* (%)	104 (25)
Diabetes mellitus, *n*	50
COPD, *n*	20
Myocardial infarction, *n*	12
Cerebrovascular accident or transient ischemic attack, *n*	11
Peripheral vascular disease, *n*	7
Liver disease, *n*	7
Peptic ulcer disease, *n*	7
Kidney disease, *n*	7
Connective tissue disease, *n*	6
Heart failure, *n*	2
History of thromboembolism, *n* (%) ^d^	57 (14)
Laboratory tests	
Hemoglobin (mmol/L) < 7.45, *n* (%)	221 (53)
White blood cell count (10^9^/L) > ULN, *n* (%)	62 (15)
Lymphocytes (10^9^/L) < LLN, *n* (%) ^e^	70 (17)
Neutrophils (10^9^/L) > ULN, *n* (%) ^f^	56 (14)
Neutrophils-to-lymphocytes (NTL) ratio > 5, *n* (%) ^g^	154 (38)
Platelets (10^9^/L) > 440, *n* (%) ^h^	30 (7)
Sodium (mmol/l) < LLN, *n* (%) ^i^	28 (7)
Creatinine clearance (ml/min/1.73 m^2^) < 60, *n* (%) ^j^	56 (14)
Albumin (g/L) < 35, *n* (%) ^k^	125 (30)
Lactate dehydrogenase (U/L) > 600, *n* (%) ^l^	34 (11)
AST (U/L) > ULN, *n* (%) ^m^	132 (32)
ALT (U/L) > ULN, *n* (%) *^n^*	59 (14)
Alkaline phosphatase (U/L) > 2.5 x ULN, *n* (%) ^o^	67 (16)

a = *n* − 7, b = *n* − 2, c = *n* − 2, d = *n* − 1, e = *n* − 3, f = *n* − 3, g = *n* − 5, h = *n* − 1, i = *n* − 2, j = *n* − 3, k = *n* − 3, l = *n* − 107, m = *n* − 8, o = *n* − 2, *p* = *n* − 2, SD = standard deviation, *n* = number of patients, ECOG/WHO = Eastern Cooperative Oncology Group/World Health Organization, BMI = body mass index, COPD = chronic obstructive pulmonary disease, ULN = upper limit of normal, LLN = lower limit of normal, AST = aspartate aminotransferase, ALT = alanine aminotransferase.

**Table 2:** Reasons for early trial discontinuation.

Characteristic	*n* (%)
Early trial discontinuation	82 (20)
Reasons for early trial discontinuation (*n* = 82) ^a^	
Progressive disease	36 (44)
Physical deterioration (not otherwise specified)	15 (18)
Patient’s request	10 (12)
Toxicity (not dose limiting)	10 (12)
Dose-limiting toxicity or serious adverse event	5 (6)
Death	4 (5)
Protocol violation	1 (1)
90-day mortality ^b^	88 (22)

a = *n* − 1, b = *n* − 19.

**Table 3:** Results of the univariable, multivariable and logistic regression analyses for early trial discontinuation.

Predictor	Univariable Analysis		Multivariable Analysis	
	OR (95%CI)	*p*	OR (95%CI)	*p*
ECOG/WHO performance status				
1 vs. 0	**2.98 (1.51–5.86)**	**<0.01**	**2.07 (1.03–4.19)**	**0.04**
BMI (kg/m^2^)	0.96 (0.92–1.01)	0.14		
<18.5 vs. ≥18.5	**2.92 (1.15–7.41)**	**0.02**		
Use of opioids vs. non-use	**2.44 (1.48–4.01)**	**<0.01**	**1.82 (1.07–3.08)**	**0.03**
Metastatic sites				
≥3 metastatic sites vs. <3	1.45 (0.89–2.35)	0.14		
Charlson Comorbidity Score				
≥1 vs. 0	0.74 (0.41–1.33)	0.31		
History of thromboembolism				
Yes vs. no	1.23 (0.63–2.42)	0.54		
Laboratory tests				
Hemoglobin (mmol/L) < 7.45	1.58 (0.96–2.59)	0.07		
White blood cell count (10^9^/L) > ULN	1.67 (0.90–3.11)	0.10		
Lymphocytes (10^9^/L) < LLN	1.12 (0.60–2.10)	0.73		
Neutrophils (10^9^/L) > ULN	**2.20 (1.18–4.11)**	**0.01**		
Neutrophils-to-lymphocytes (NTL) ratio > 5	1.52 (0.93–2.49)	0.09		
Platelets (10^9^/L) > 440	**2.55 (1.16–5.60)**	**0.02**		
Sodium (mmol/L) < LLN	**4.04 (1.84–8.88)**	**<0.01**	**2.95 (1.27–6.84)**	**0.01**
Creatinine clearance (mL/min/1.73 m^2^) < 60	1.00 (0.49–2.03)	1.00		
Albumin (g/L) < 35	**2.56 (1.55–4.23)**	**<0.01**		
Lactate dehydrogenase (U/L) > 600	**2.25 (1.03–4.93)**	**0.04**		
AST (U/L) > ULN	**2.22 (1.34–3.68)**	**<0.01**		
ALT (U/L) > ULN	1.33 (0.69–2.57)	0.39		
Alkaline phosphatase (U/L) > 2.5 × ULN	**3.36 (1.90–5.93)**	**<0.01**	**2.72 (1.49–4.99)**	**0.001**

OR = odds ratio, CI = confidence interval, ECOG/WHO = Eastern Cooperative Oncology Group/World Health Organization, BMI = body mass index, ULN = upper limit of normal, LLN = lower limit of normal, AST = aspartate aminotransferase, ALT = alanine aminotransferase. Bold: Significant.

## Data Availability

The data presented in this study are available on request from the corresponding author. The data are not publicly available due to confidentiality rules of the original phase I clinical trials.

## Appendix A

**Table A1:** Characteristics of included trials.

EUdraCT/NCT Trial Number:	Title	Population	Participants, *n*
NCT02058901	High-dose protein kinase inhibitor	Solid tumors	81
NCT02636426	High-dose protein kinase inhibitor	Solid tumors	18
2011–005116-28	Cytotoxic drug in combination with radiopharmaceutical	Prostate cancer	6
2014–000201-12	Bispecific antibody (CEA antigen and CD3 T-cell)	CEA-expressing gastrointestinal adenocarcinomas	4
2015–000673-12	DNA-dependent protein kinase inhibitor	Solid tumors	4
2011–002713-10	Antibody-drug conjugate (anti-mesothelin—antimitotic agent)	Unresectable pancreatic cancer and platinum-resistant ovarian cancer	5
2013–003041-41	Targeted immunocytokine (CEA-IL2v)	Solid tumors	11
2013–002663-25	Vaccine (hVEGF_26–104_/RFASE)	Solid tumors	25
NCT00989651	A Phase I Study of Intravenous Carboplatin/Paclitaxel or Intravenous and Intraperitoneal Paclitaxel/Cisplatin in Combination with Continuous or Intermittent ABT-888 and Bevacizumab in Newly Diagnosed Patients with Previously Untreated Epithelial Ovarian, Fallopian Tube or Primary Peritoneal Cancer.	Epithelial ovarian, fallopian tube or primary peritoneal cancer.	1
NCT01366144	An Early Phase 1 Study of ABT-888 in Combination with Carboplatin and Paclitaxel in Patients with Hepatic or Renal Dysfunction and Solid Tumors.	Solid tumors	1
NCT01351103	A Phase I, open-label, dose escalation study of oral LGK974 in patients with malignancies dependent on Wnt ligands	Advanced solid tumors	2
NCT01587703	A Phase I/II Open-Label, Dose Escalation Study to Investigate the Safety, Pharmacokinetics, Pharmacodynamics, and Clinical Activity of GSK525762 in Subjects with NUT Midline Carcinoma (NMC) and Other Cancers	NUT midline carcinoma (NMC) and other cancers	1
NCT01693562	A Phase 1/2 Study to Evaluate the Safety, Tolerability, and Pharmacokinetics of MEDI4736 in Subjects With Advanced Solid Tumors.	Advanced solid tumors	1
NCT01783171	A Phase I Trial of Dinaciclib (SCH727965) and MK2206 in Metastatic Pancreatic Cancer with an Expansion Cohort in Advanced Pancreatic Cancer	Advanced and metastatic pancreatic cancer	2
NCT01968109	A Phase 1/2a Dose Escalation and Cohort Expansion Study of the Safety, Tolerability, and Efficacy of Anti-LAG-3 Monoclonal Antibody (BMS-986016) Administered Alone and in Combination with Anti-PD-1 Monoclonal Antibody (Nivolumab, BMS-936558) in Advanced Solid Tumors	Advanced solid tumors	27
NCT02048384	An Exploratory Study of Metformin With or Without Rapamycin as Maintenance Therapy After Induction Chemotherapy in Subjects with Metastatic Pancreatic Adenocarcinoma	Pancreatic cancer	7
NCT01749397	A Phase I Trial of the Combination of the PARP Inhibitor ABT-888 with Intraperitoneal Floxuridine (FUDR) in Epithelial Ovarian, Primary Peritoneal and Fallopian Tube Cancers	Epithelial ovarian, primary peritoneal and fallopian tube cancers	2
NCT02073994	A Phase 1, Multicenter, Open-Label, Dose-Escalation and Expansion, Safety, Pharmacokinetic, Pharmacodynamic, and Clinical Activity Study of Orally Administered AG-120 in Subjects with Advanced Solid Tumors, Including Glioma, with an IDH1 Mutation.	Advanced solid tumors	3
NCT02248805	A Phase 1, First-in-Human, Open Label, Dose Escalation Study of MGD007, A Humanized gpA33 x CD3 Dual-Affinity Re-Targeting (DART^®^) Protein in Patients with Relapsed/Refractory Metastatic Colorectal Carcinoma	Advanced colorectal cancer	10
NCT02000947	A Phase 1b Open-label Study to Evaluate the Safety and Tolerability of MEDI4736 in Combination with Tremelimumab in Subjects with Advanced Non-small Cell Lung Cancer.	Advanced non-small-cell lung cancer	3
NCT02035358	A Phase 1 Study of HyperAcute Renal (HAR) Immunotherapy In Patients with Metastatic Renal Cell Cancer	Advanced renal cell cancer	1
NCT02501902	An open-label phase IB study of Palbociclib (oral CDK 4/6 inhibitor) plus Abraxane (Nab-paclitaxel) in patients with metastatic pancreatic ductal adenocarcinoma	Advanced pancreatic cancer	2
NCT02625857	An Open-Label, Phase 1 Study of the Safety and Immunogenicity of JNJ-64041809, a Live Attenuated Listeria monocytogenes Immunotherapy, in Subjects with Metastatic Castration-resistant Prostate Cancer	Prostate cancer	5
NCT02592967	An Open-Label, Phase 1 Study of the Safety and Immunogenicity of JNJ-64041757, a Live Attenuated Listeria monocytogenes Immunotherapy, in Subjects With Non-Small Cell Lung Cancer	Non-small-cell lung cancer	5
NCT02319018	A Phase 1 Study of alisertib (MLN8237) in combination with mFOLFOX in gastrointestinal tumors	Gastrointestinal tumors	4
NCT02514031	Phase I/Ib, single-arm, open-label, multi-center trial using ARQ-761 (beta-lapachone) treatment with gemcitabine/nab-paclitaxel chemotherapy in metastatic, unresectable, or recurrent pancreatic cancer	Advanced pancreatic cancer	6
NCT02766699	A Phase 1 study to evaluate the safety, tolerability, and immunogenicity of EGFR (Vectibix^®^ sequence)-targeted EDVs containing doxorubicin (EGFR(V)-EDV-Dox) in subjects with recurrent glioblastoma multiforme (GBM)	Recurrent glioblastoma	6
NCT02716948	A Pilot Study of Stereotactic Radiosurgery combined with Nivolumab in Patients with Newly Diagnosed Melanoma Metastases in the Brain and Spine	Melanoma	4
NCT02588443	Phase I study of neo-adjuvant RO7009789 alone or neo-adjuvant RO7009789 plus nab-paclitaxel and gemcitabine followed by adjuvant RO7009789 plus nab-paclitaxel and gemcitabine for patients with newly diagnosed resectable pancreatic carcinoma.	Pancreatic cancer	3
NCT02453620	A Phase 1 Study Evaluating Safety, Tolerability, and Preliminary Antitumor Activity of Entinostat and Nivolumab with or without Ipilimumab in Advanced Solid Tumors	Advanced solid tumors	12
NCT02572687	An Open-Label, Multicenter, Phase 1 Study of Ramucriumab Plus MEDI4736 in Patients with Locally Advanced and Unresectable or Metastatic Gastrointestinal or Thoracic Malignancies	Advanced gastrointestinal or thoracic malignancies	1
NCT01804530	A Phase 1 Study to Assess Safety, Pharmacokinetics, and Pharmacodynamics of PLX7486-TsOH as a Single Agent and in Combination with Gemcitabine and nab-Paclitaxel in Patients with Advanced Solid Tumors.	Advanced solid tumors	4
NCT02512172	A study of using epigenetic modulators to enhance response to MK-3475 in microsatellite stable advanced colorectal cancer	Colorectal cancer	16
NCT02491411	A Pilot Study of Dexamethasone Therapy Prior to Rechallenge with Enzalutamide in Men with Metastatic Castration-Resistant Prostate Cancer Dex EXTends Enza Response (The DEXTER Trial)	Prostate cancer	1
NCT02358473	Open-label, Multicenter Phase 1 Study of Mogamulizumab (KW-0761) in Combination with Docetaxel in Previously Treated Subjects with Non-small Cell Lung Cancer (NSCLC)	Non-small-cell lung cancer	1
NCT02355535	Phase I Study of Procaspase Activating Compound-1 (PAC-1) in the Treatment of Advanced Malignancies	Advanced solid tumors	7
NCT02262741	A Phase 1 Study to Evaluate the Safety, Tolerability, and Efficacy of MEDI4736 in Combination with Tremelimumab in Subjects with Recurrent or Metastatic Squamous Cell Carcinoma of the Head and Neck.	Advanced head and neck cancer	1
NCT02619253	A Phase I/Ib, Open Label, Dose Finding Study to Evaluate Safety, Pharmacodynamics and Efficacy of Pembrolizumab (MK-3475) in Combination with Vorinostat in Patients with Advanced Renal or Urothelial Cell Carcinoma	Advanced renal or urothelial cell carcinoma	11
NCT02890069	Phase Ib, open-label, multi-center study to characterize the safety, tolerability and pharmacodynamics (PD) of PDR001 in combination with LCL161, everolimus (RAD001) or panobinostat	Advanced solid tumors	10
NCT02631733	A Phase I Study of a Combination of MM-398 and Veliparib in Solid Tumors	Advanced solid tumors	2
NCT02833883	A Phase 1b Study of Enzalutamide plus CC-115 in Men with Castration-Resistant Prostate Cancer (CRPC)	Prostate cancer	5
NCT02989636	Phase I Safety Study of Stereotactic Radiosurgery with Concurrent and Adjuvant PD-1 Antibody Nivolumab in Subjects with Recurrent or Metastatic Chordoma	Advanced chordoma	3
NCT03043989	Two independent phase 1b cohorts of docetaxel or cabazitaxel in combination with the potent CYP3A4 inhibitor, clarithromycin.	Prostate cancer	4
NCT02655822	A Phase 1/1b, Open-Label, Multicenter, Repeat-Dose, Dose-Selection Study of CPI-444 as Single Agent and in Combination with Atezolizumab in Patients with Selected Incurable Cancers	Advanced solid tumors	14
NCT02819999	A Study of Rovalpituzumab Tesirine (SC16LD6.5) in the Frontline Treatment of Patients with Extensive Stage Small Cell Lung Cancer	Small-cell lung cancer	1
NCT02520778	9903 A Phase 1B Study of AZD9291 in Combination with Navitoclax in EGFR-Mutant Non-Small Cell Lung Cancer Following Resistance to Initial EGFR Kinase Inhibitor	Non-small-cell lung cancer	1
NCT03051477	A Phase I Dose Escalating Trial of Mistletoe Extract in Patients with Advanced Solid Tumors	Advanced solid tumors	12
NCT02608125	A Phase 1 Open-Label, Multicenter, Dose-Escalation Study of PRN1371, a FGFR1–4 Kinase Inhibitor, in Adult Patients with Advanced Solid Tumors, followed by an Expansion Cohort in Patients with FGFR1, 2, 3, or 4 Genetic Alterations	Advanced solid tumors	2
NCT02722538	A Phase 1b, Multicenter, Open Label Study Evaluating Safety, Tolerability and Preliminary Efficacy of GemRIS 225 mg in Subjects with Muscle-Invasive Transitional Cell Carcinoma of the Bladder	Bladder cancer	1
NCT02903914	Safety, Pharmacokinetics, and Pharmacodynamics of Escalating Oral Doses of the Arginase Inhibitor INCB001158 (formerly known as CB-1158) as a Single Agent and in Combination with Immune Checkpoint Therapy in Patients with Advanced/Metastatic Solid Tumors	Advanced solid tumors	8
NCT03246074	Phase I Clinical Trial of Combined Fostamatinib and Paclitaxel in Ovarian Cancer	Ovarian cancer	3
NCT03148418	An Open-Label, Multicenter Extension and Long-term Observational Study in Patients Previously Enrolled in a Genentech and/or F. Hoffman-La Roche LTD Sponsored Atezolizumab Study	Advanced solid tumors	1
NCT3238027	A Phase 1, Open-Label, Dose Escalation Trial to Investigate the Safety, Tolerability, Pharmacokinetics and Pharmacodynamic Activity of SNDX-6352 Monotherapy and SNDX-6352 in Combination with Durvalumab in Patients with Unresectable, Recurrent, Locally-Advanced, or Metastatic Solid Tumors	Advanced solid tumors	10
NCT03219268	A Phase 1, First-in-Human, Open-Label, Dose Escalation Study of MGD013, A Bispecific DART^®^ Protein binding PD-1 and LAG-3 in Patients with Unresectable or Metastatic Neoplasms	Advanced solid tumors	6
NCT03058289	A Phase 1/2 Safety Study of Intratumorally Administered INT230–6 in Adult Subjects with Advanced Refractory Cancers	Advanced solid tumors	2
NCT03299946	Feasibility and Efficacy of Neoadjuvant Cabozantinib plus Nivolumab (CaboNivo) Followed by Definitive Resection for Patients with Locally Advanced Hepatocellular Carcinoma (HCC)	Hepatocellular carcinoma (HCC)	3
NCT02675946	A Phase 1 Open-label Dose Escalation and Dose Expansion Study of CGX1321 in Subjects with Advanced Solid Tumors and Phase 1b Study of CGX1321 in Combination with Pembrolizumab in Subjects with Advanced Gastrointestinal Tumors	Advanced gastrointestinal tumors	3
NCT03257761	A Phase Ib Study of Guadecitabine (SGI-110) and Durvalumab (MEDI 4736) in Patients with Advanced Hepatocellular Carcinoma, Pancreatic Adenocarcinoma, and Cholangiocarcinoma/Gallbladder Cancer	Advanced hepatocellular carcinoma, pancreatic adenocarcinoma and cholangiocarcinoma/gallbladder cancer	1
NCT02900664	Phase Ib, open-label, multi-center study to characterize the safety, tolerability and pharmacodynamics (PD) of PDR001 in combination with CJM112, EGF816, Ilaris^®^ (canakinumab) or Mekinist^®^ (trametinib)	Advanced solid tumors	1
2437136	A Phase 1b/2, Open-label, Dose Escalation Study of Entinostat in Combination with Pembrolizumab in Patients with Non-small Cell Lung Cancer, with Expansion Cohorts in Patients with Non-small Cell Lung Cancer, Melanoma and Mismatch Repair-Proficient Colorectal Cancer	Non-small-cell lung cancer, melanoma, mismatch repair-proficient colorectal cancer	6
NCT03138538	An Open-label, Phase I, Dose Escalation Trial of Methionine Aminopeptidase 2 Inhibitor M8891 in Subjects with Advanced Solid Tumors	Advanced solid tumors	3
3137888	Pilot study of spectroscopic MRI-guided, dose-escalated radiation therapy for newly-diagnosed glioblastoma	Glioblastoma	1
NCT01849146	Phase I Study of AZD1775 (adavosertib) with Radiation and Temozolomide in Patients with Newly Diagnosed Glioblastoma and Evaluation of Intratumoral Drug Distribution in Patients with Recurrent Glioblastoma	Recurrent glioblastoma	1
NCT02575794	Phase 1 Dose Escalation and Drug Distribution Study of Oral Terameprocol in Patients with Recurrent High-Grade Glioma	High-grade glioma	2
NCT02658981	A Phase I Trial of Anti-LAG-3 or Anti-CD137 Alone and in Combination with Anti-PD-1 in Patients with Recurrent Glioblastoma	Recurrent glioblastoma	3
